# A Rare Presentation of Type-III Takayasu’s Arteritis in a 20-Year-Old Female

**DOI:** 10.7759/cureus.17991

**Published:** 2021-09-15

**Authors:** Nimra Mazhar, ANUM ARIF, Ahsin Manzoor Bhatti, Bismah Riaz, Syed Hashim Ali Inam, Nawabzada Zeerak Farhat Sherwani

**Affiliations:** 1 Medicine, Combined Military Hospital, Lahore, PAK; 2 Vascular Surgery, CMH Lahore Medical College and Institute of Dentistry, Lahore, PAK; 3 Internal Medicine, CMH Lahore Medical College and Institute of Dentistry, Lahore, PAK; 4 Internal Medicine, Army Medical College, Rawalpindi, PAK; 5 Neurology, Marshall University Joan C. Edwards School of Medicine, Huntington, USA; 6 Internal Medicine, CMH Lahore Medical and Dental College, Lahore, PAK

**Keywords:** inflammation, vasculitis, pulse, claudication, syncope

## Abstract

Takayasu’s arteritis is a rare chronic granulomatous vasculitis that predominantly affects the aorta and its branches. It is estimated to affect 2.6/million/annum, predominantly women in the second or third decade of their lives. This case report describes the case of a 21-year-old female, who initially only had low-grade fever and acrodynia, which over a few months, progressed to arm and leg claudication, weight loss, nausea, headache, and dizziness. A year later, the patient experienced impalpable radial pulses bilaterally, and her CT angiogram revealed multi-level arterial stenosis. A diagnosis of Takayasu’s arteritis was made and the patient was started on steroids and methotrexate. A consult was made with vascular surgery but no intervention was deemed necessary and the patient responded well to the medical treatment given. Though Takayasu’s arteritis is a very rare disease, a detailed history, clinical examination, and investigations can help with early diagnosis.

## Introduction

Takayasu’s arteritis is a rare chronic inflammatory large vessel vasculitis and has an incidence of 2.6/million/year [1,2]. It usually affects people in the second and third decade of life, predominantly women (ratio of 4:1, female to male) [3]. Patients might present with non-specific symptoms or with characteristic features depending upon the site of the arterial lesion [1]. It is most commonly seen in Japan, South East Asia, India, and Mexico [1]. This report describes the case of a 21-year-old female who was diagnosed to have Takayasu arteritis.

## Case presentation

A 21-year-old female with no comorbidities and negative family history for any autoimmune disease started experiencing low-grade undocumented fever for 3 months in August 2019 which was continuous, non-radiating, and shooting in nature with no aggravating or relieving factors. Radiological studies were unremarkable.

Over the course of a few months, the patient began to experience arm and limb claudication. This lasted 30 days and was relieved on its own, only to reoccur 10 months later. During this period, the patient also suffered a 5-kilogram weight loss, headache, dizziness, and nausea. She visited a local general practitioner, who offered her symptomatic treatment. 

**Figure 1:**
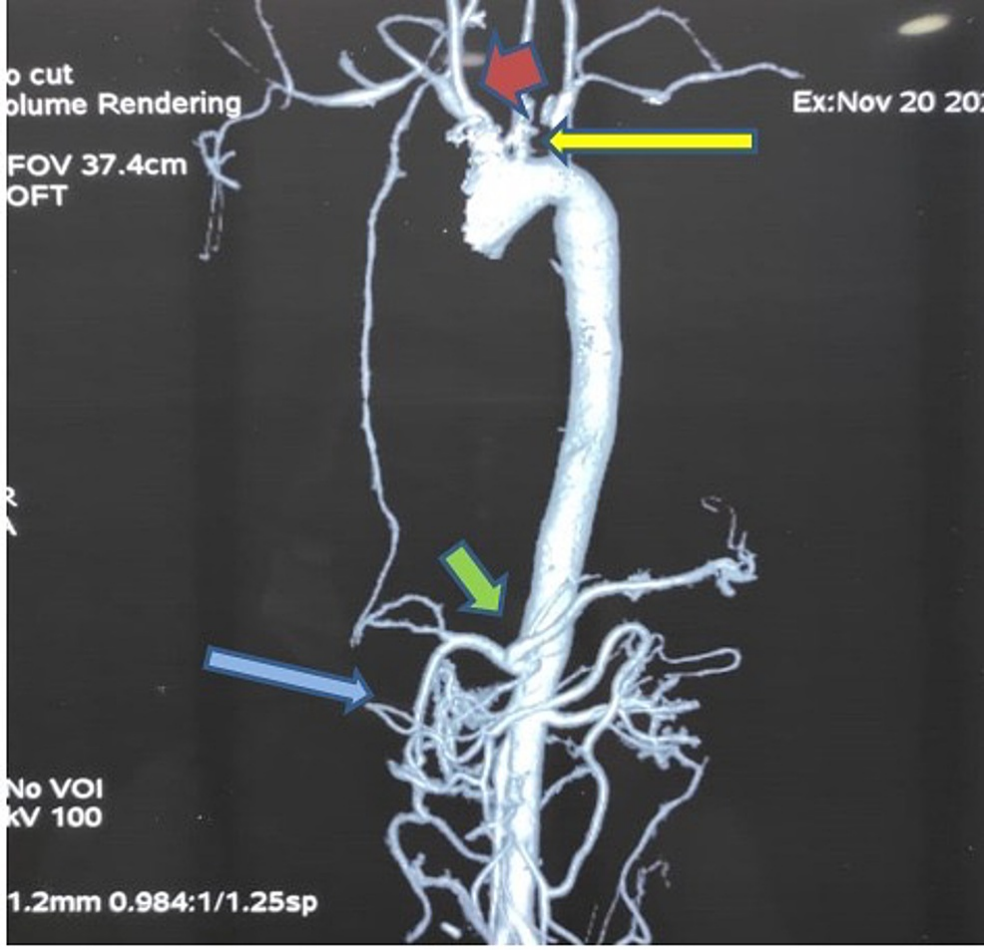
CT angiogram coronal view. The red arrow shows narrowing of the right internal carotid artery (a), the yellow arrow indicates narrowing of the origin of the right subclavian artery (b), the green arrow indicates narrowing of the origin of celiac trunk (c), and the blue arrow indicates the narrowed origin of the superior mesenteric artery, and its beaded appearance (d)

In October of 2020, the patient noticed that her radial pulses were weak and she was admitted. On examination, her brachial and radial pulses were weak, impalpable bilaterally and her BP was non-recordable manually. Ophthalmic examination revealed bilateral narrowing of arterioles in both eyes. However, no sign of arteritis was observed in the retina. Investigations indicated that the patient had hypochromic microcytic anaemia with haemoglobin level of 8. Furthermore, a CRP of 40, an ESR of 50, and a low HDL cholesterol of 30 were observed. CT angiogram showed narrowing in right ICA and left CCA, a segment of the right subclavian artery was narrowed distal to its origin from brachiocephalic artery, short segment narrowing in the celiac trunk, and narrowing at origin of SMA as can be appreciated in Figure 1.

Along with this, left superficial and deep femoral arteries were markedly attenuated with collateral formation. The rest of the investigations including ANA and anti-ENA screens were negative. The patient was started on methylprednisolone 500 mg IV for 3 days, tablet prednisone 25mg BD, tablet aspirin 75 mg OD, tablet methotrexate 20g weekly, capsule gabapentin 75 mg BD, tablet folic acid 5 mg OD, and tablet atorvastatin 20 mg HS. The patient responded well to the treatment given, her pulses were palpable again and her symptoms improved. Inflammatory markers came down to normal range and steroids were gradually tapered off over a period of 1 month. Currently patient remains asymptomatic.

## Discussion

Takayasu’s arteritis is also known as giant cell arteritis or pulseless disease [4]. Although the etiology is unknown, Takayasu arteritis causes granulomatous inflammation resulting in stenotic or occlusive lesions of large vessels [5]. The most commonly affected vessel is the subclavian, followed by common carotid, brachiocephalic and vertebral arteries [5]. It is usually considered a tri-phasic disease with the first stage characterized by systemic inflammation. The second stage results in arterial injury, and the third stage being the fibrotic burnt-out phase [5]. In adults, approximately 80% of patients of Takayasu’s arteritis are women with a female to male ratio being 9:1 from Japan; the current case report is also that of a female [4].

Takayasu’s arteritis may result in non-specific symptoms of upper body pain, intermittent claudication, headache, dizziness, syncope, and visual deterioration [6]. Involvement of aortic branches can result in malaise, headache, decreased to absent pulses of upper extremities, dysfunction of upper extremities, dizziness, visual disturbances, and syncope [4]. Of the above mentioned, our patient experienced all excluding syncope and visual disturbances. As the initial symptoms are non-specific, diagnosis is frequently delayed, often resulting in complications later in the course of diagnosis. One study reported a patient presenting with acute myocardial ischemia [6]. Our patient was diagnosed as a case of Takayasu’s arteritis based upon criterion proposed by the American college of rheumatology, of which our patient had four i.e. onset before 40 years, claudication of extremities, decrease in brachial pulses, and narrowing of the carotid artery [3,4].

We compare our case to a documented case of Takayasu's arteritis which describes a 54-year-old female who presented with acute pulmonary edoema and non-specific symptoms of fever, fatigue, and weakness since the age of 35 [7]. Our patient, also a female, began having similar non-specific symptoms at the age of 20 years, but she also had discomfort in the neck, arm, and leg claudication. Our patient had identical bilateral outcomes to that case, with undetectable blood pressure and impalpable radial and brachial pulses in the left arm [7]. Investigations of both cases revealed similar findings [7]. CXR of the compared case indicated bilateral infiltrates, and angiography revealed focal narrowing of the thoracic and abdominal aortas, as well as blockage of both subclavian arteries and the right coronary artery [7]. Our patient's angiography likewise demonstrated multi-level arterial stenosis. Two stents were placed in the marginal obtuse vessal of the compared case and she was started on methylprednisolone, clopidogrel, and furosemide [7]. Our patient was likewise treated along the same lines, however, she was also given methotrexate.

In our institution, we recently published a case report in which the patient's diagnosis of Takayasu's arteritis was also delayed, resulting in complications [8]. This was the case of a 33-year-old man who presented to the emergency department multiple times for a range of symptoms, including fever, myalgias, left arm numbness, and persistent hypertension [8]. His CT aortogram revealed multi-vessel narrowing, including the celiac axis, superior mesenteric, renal, and internal iliac arteries, as well as an atrophied right kidney [8]. Despite being put on corticosteroids and immunosuppressants, the patient developed peritonitis after five years, and a CT angiography of the abdomen revealed stenosis of the inferior mesenteric artery and left renal artery [8]. Vascular surgeons were consulted and stenting of the left renal and inferior mesenteric arteries was planned. Our patient was treated on similar lines and she responded well. Moreover, vascular surgeons were consulted but no intervention was deemed necessary.

Takayasu’s arteritis is a disease with a wide range of non-specific early and late symptoms that are frequently missed by physicians. Most people do not seek medical attention until serious complications have occurred. We lack specialised vascular surgery centres in Pakistan since we are a developing country with a recent introduction of vascular surgery. Patients are unclear where to present themselves, and even general practitioners are hesitant to refer such patients to the clinic. Although our patient's symptoms were typical, they were nonspecific and difficult to identify by a clinician unfamiliar with the vascular field. Therefore, due to the lack of a national data registry, we are unable to examine the outcomes of similar cases in our country. Nevertheless, by publishing it here, we want to take the initiative and set benchmarks.

## Conclusions

Takayasu’s arteritis is a rare disease and in most cases, the diagnosis gets delayed. Therefore, even vague symptoms like malaise, headache, and fatigue should be thoroughly investigated, and patients should be examined properly so signs like impalpable pulses and discrepancy in BP can be picked up early. If we diagnose this disease early, we can help decrease morbidity and mortality due to the patients presenting late with complications like acute MI, pulmonary edema, and visual deterioration.

## References

[1] Lambert M, Hachulla E, Huglo D, Hatron PY (2009). Takayasu arteritis: a review. Med Nucl.

[2] Liu H, Sun L, Upadhyaya RS, Chen Y, Ajoje OO (2018). Case report: Takayasu arteritis in a 3-month-old Chinese girl. Medicine (Baltimore).

[3] Lumbreras-Marquez J, Castillo-Reyther RA, De-la-Maza-Labastida S, Vazquez-Alaniz F (2018). Takayasu arteritis a cause of hypertensive disorder of pregnancy: a case report. J Med Case Rep.

[4] Khan M, Banoo H (2013). A case report of Takayasu's arteritis. Med Today.

[5] Verweij KE, Van Well AME, Vd Sluijs JW, Dees A (2012). Late onset takayasu arteritis and rheumatoid arthritis. Case Rep Med.

[6] Zhang T, Peng B, Tu X, Zhang S, Zhong S, Cao W (2019). Acute myocardial infarction as the first manifestation of Takayasu arteritis: a case report. Medicine (Baltimore).

[7] Manfrini O, Bugiardini R (2006). Takayasu's arteritis: a case report and a brief review of the literature. Heart Int.

[8] Khan R, Arif A, Inam SH, Riaz B, Jamil H (2021). Takayasu's arteritis in a 33-year-old male. Cureus.

